# *Helicobacter pylori* Infection, Chronic Inflammation, and Genomic Transformations in Gastric MALT Lymphoma

**DOI:** 10.1155/2013/523170

**Published:** 2013-03-28

**Authors:** Magdalena Witkowska, Piotr Smolewski

**Affiliations:** Department of Experimental Hematology, Medical University of Lodz, Ciolkowskiego 2, 93-510 Lodz, Poland

## Abstract

Nowadays, it is believed that the main role in the development of gastric mucosa-associated lymphoid tissue (MALT) lymphoma plays *Helicobacter pylori* infection. This world-wide distributed bacteria is in charge of most cases of not only upper gastrointestinal tract disorders but also some of extragastric problems. Constant stimulation of the immune system causes a B-lymphocytes proliferation, which is considered to be responsible for the neoplastic transformation. On the other hand, there are 10%–20% of patients who do not respond to *Helicobacter pylori* eradication treatment. This group has often a chromosome translocation, which suggests that there is another unknown, so far, pathogenetic mechanism of MALT lymphoma. Majority of genetic abnormalities are connected with nuclear factor-**κ**B (NF-**κ**B) pathway, which activates the uncontrolled proliferation of neoplastic cells. Translocations already described in studies are t(11;18)(q21;q21), which is the most common, t(14;18)(q32;q21), t(14;18)(q32;q21), and t(3;14)(p14.1;q32). This non-Hodgkin's lymphoma is an indolent type originated outside lymph nodes. In more than 50% of cases, it occurs in the stomach. Occasionally, it can be found in salivary and thyroid gland, lung, breast, bladder, skin, or any other place in the human body. This paper is a review of the current knowledge on etiology, pathogenesis, treatment, and follow-up of gastric MALT lymphoma.

## 1. Introduction

The name of mucosa-associated lymphoid tissue (MALT) lymphoma was first established in 1983 by Isaacson and Du [1]. From the beginning, it was adopted well and is still used in an unchanged form. Marginal zone lymphoma of MALT is, apart from diffuse large B-cell lymphoma, the most frequent type of lymphoma that occurs in the stomach. What is important is that it can develop in almost every organ and tissue, for instance lungs, breast, thyroid gland, bladder, skin, or orbital adnexa. It is an indolent type, but clinical outcomes and response to treatment vary among patients. MALT lymphoma arises from the extranodal sites reach in B-lymphocytes, which appears in response to chronic antigenic stimulation caused by infection (*Helicobacter pylori*) or autoimmune process (Hashimoto disease). This disorder is the best example of how infectious pathogens and genetic abnormalities lead to malignant transformation. Gastric MALT lymphoma pathogenesis is a complex process including many gene alternations that result in cancer appearance. Better understanding of the background of the disease is crucial for discovering new prognostic factors, helpful in deciding when more aggressive treatment should be employed.

## 2. Epidemiology

The incidence of malignant lymphomas is at the rate of 3%-4% of all malignancy worldwide and has been increasing during the last 50 years. Lately, some stabilization in the number of diagnosis was observed, but only in developed countries. Malignant lymphomas are observed to be more frequent in North America, Australia, and Europe than in Asia and Africa. MALT lymphomas determine almost 7% of all non-Hodgkin's lymphoma, and at least 40% is primarily located in stomach. It is confirmed that gastric MALT lymphoma occurs in younger patients than the rest of malignant lymphomas. The MALT lymphoma is mainly a disease of older adults, with a median age of 60 years. There is a gentle predominance of females [2]. In Asia, there is much higher proportions of MALT lymphomas, which can be caused by more frequent prevalence of *Helicobacter pylori* in this region of the world. 

## 3. Pathogenesis

### 3.1. Infectious Background

Gastric MALT lymphoma pathogenesis is strictly connected with *Helicobacter pylori* infection. Although 90% of population worldwide have confirmed bacteria colonization, only 2% will develop malignant lymphoma. It was confirmed by Weber et al. [3] that almost 90% of patients with gastric MALT lymphoma are infected with *Helicobacter pylori*. This curved bacillus, previously called *Campylobacter pyloridis*, is a Gram negative pathogen found in the stomach. It was discovered by Marshall and Warren in 1980s [4]. From the beginning, *Helicobacter pylori* was classified as a higher class I carcinogen. Although over 80% of people are asymptomatic, chronic infection can lead to gastritis, gastric and duodenal ulcer, gastric adenocarcinoma, and MALT lymphoma [5, 6]. Nowadays, it is widely accepted that *Helicobacter pylori* gastritis is crucial in an evolution of MALT lymphoma localized in stomach. It was confirmed by several studies that chronic gastric inflammation causes constant antigenic stimulation, which leads to clonal expansion of B-cell lymphocytes [7, 8].

**Figure 1:**
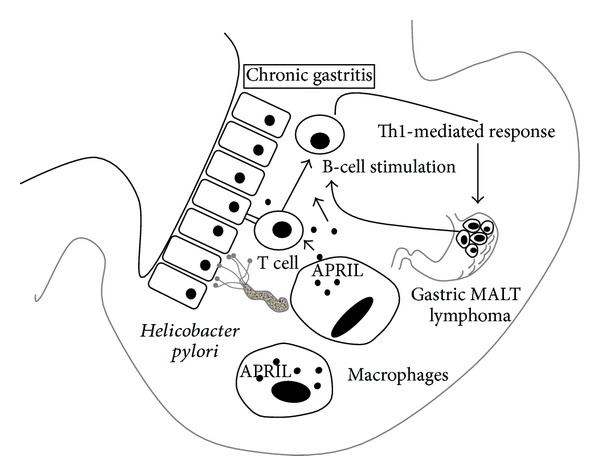
*Helicobacter pylori*-caused gastritis is crucial in an evolution of gastric MALT lymphoma localized in the stomach. In the gastric mucosal cells, there are elevated levels of some cytokines, including proliferation-inducing ligand (APRIL), the protein with a crucial role in B-cell maturation and survival. APRIL induces also B-cells transformation and the lymphoma progression. Gastritis attracted macrophages, which, under a *Helicobacter pylori* infection, release large amounts of APRIL.

In the gastric mucosal cells, there are elevated levels of some cytokines, including proliferation-inducing ligand (APRIL), which belongs to the tumour necrosis factor (TNF) family. The protein has a crucial role in B-cell maturation and survival. APRIL is produced by macrophages present in the gastric MALT infiltrate, located close to the neoplastic cells [9]. APRIL may also induce B-cells transformation and the progression to the diffuse large B-cell lymphoma (Figure 1). The survival and transformation of B cells in malignant lymphoma require additional signals. They come either from T cells or directly by the antigenic autostimulation of lymphoma cells [1]. Gastric inflammation causes the appearance of a large number of macrophages, which, under a *Helicobacter pylori* infection, release large amounts of APRIL. This mechanism may be enhanced and maintained by the activated T lymphocytes. Importantly, a number of APRIL-producing macrophages significantly decrease in complete remission after eradication therapy [9]. Thus, a new APRIL production-targeted therapy can be developed.

Other pathogens, are also suspected to play an important role in MALT lymphoma pathogenesis. There are bacteria such as *Campylobacter jejuni*, *Borrelia burgdorferi*, and *Chlamydia psittaci* and viruses like Hepatitis C virus (HCV) that are potentially responsible for oncogenesis. These pathogens were found in histological material, but so far no strong evidences were established [10].

### 3.2. Autoimmune Disease

Patients with autoimmune disease have for sure higher risk of developing MALT lymphoma. Autoreactive B cells infiltrate the healthy organs and create lymphoid infiltrate similar to normal MALT tissue with huge amount of reactive clonal B lymphocytes. This situation is observed in salivary gland in patients with diagnosis of Sjögren syndrome and in the thyroid gland in Hashimoto disease. Sjögren syndrome is associated with 44 times increased risk of lymphoma [11], whereas Hashimoto's thyroiditis causes 70 times increased risk of thyroid lymphoma [12]. 

### 3.3. Genetic Abnormalities

Gastric MALT lymphoma is connected with many genetic abnormalities and transformations. Some of them are proven to be strongly associated with the disease, but some are still not confirmed. It is believed, that on the background of chronic inflammation not only reactive B-cells are stimulated but also activated neutrophils which can lead to production of oxygen species. As a result, this genotoxins provoke DNA damages, which are responsible for mutations and transformations of genetic material. 

The best known abnormality is t(11;18)(q21;q21), which was first described in 1989 [13]. It originates from a fusion of two proteins: apoptosis inhibitor 2 (API2) and paracaspase MALT lymphoma-translocation gene 1 (MALT1). It is extremely important that this translocation is present only in MALT lymphomas. What is more important is that while t(11;18)(q21;q21) is detected, no other chromosome abnormality can be found [14]. Unfortunately, positive cases do not response to *Helicobacter pylori* eradication, but, in contrast, they do not transform to more aggressive diffusive large B-cell lymphoma [15]. It is known that complete remission can be seen in at least 20% of patients with t(11;18)(q21;q21). The incidence of positivity for this translocation MALT lymphoma is at approximately 20% in Europe [16, 17] but is not as common in the United States where only 5% are positive [18].

Another translocation is detected in only 5% of gastric MALT lymphomas. Patients with t(1;14)(p22;q32) or its variant t(1;2)(p22;p12) often have other genomic mutations. Moreover, it is usually connected with an advanced stage of disease and poor outcomes. *BCL-10 *gene is relocated from chromosome 1 to 14, which in consequence triggers overexpression of Bcl-10 protein also known as CIPER, CARMEN, or mE10. In healthy organisms, higher expression is observed in lymph nodes, spleen, and testis. So far, it is believed that Bcl-10 protein expression is responsible for proliferative effects [19, 20]. 

The t(14;18)(q32;q21)(*IGH-BCL-2*) is commonly present in follicular lymphoma, in about 20% of diffuse large B-cell lymphoma and sometimes in chronic lymphocytic leukemia. Although this aberration is extremely rare in other types of lymphomas, it can be found in some cases of gastric MALT lymphoma. It was discovered that this aberration occurs more often in HCV-infected patients [21]. Bcl-2 is an antiapoptotic protein, which helps in survival and expansion of clonal B cells. So far, the role of t(14;18) in gastric MALT lymphoma is not fully understood. Overexpression of Bcl-2 is found not only in translocation positive patients but also in the negative ones. It is believed that similar to other types of lymphomas, *t(14;18)(IGH-BCL-2)*  must coexist with other genetic abnormalities in order to develop neoplasm.

t(3;14)(p14;q32)(*IGH-FOXP1*) is a newly described abnormality present in patients with MALT lymphoma. This aberration causes overexpression of forkhead box (FOX)P1 mRNA and protein [22]. Accurate mechanism of how transcription factor FOXP1 leads to lymphogenesis is not fully discovered. The first study showed that positivity for this translocation is approximately 10% of all MALT lymphoma patients [22]. This abnormality is commonly found with other genetic aberrations. The most recent studies described the presence of t(3;14)(p14;q32) in diffuse large B-cell lymphoma, outside the lymph nodes especially [23, 24]. Only one study, so far, confirmed the existence of this translocation in gastric MALT lymphoma [25] which involved bad clinical outcomes.

In pathogenesis of MALT lymphoma, the above described translocation promotes oncogenesis by similar well-known mechanism. The majority of them involve the same pathway, which leads to antigen receptor-mediated activation of NF*κ*B. This is a crucial transcript factor which plays a key role in MALT lymphogenesis [26, 27]. It regulates processes connected with B-cell development, growth, and survival by production of cytokines and growth factors, for example, TNF-*α* family (BAFF). Latest studies have shown that B-cell activation in MALT lymphoma can be strictly connected with TNF family. It can be also responsible for activation of cell apoptosis [28, 29]. It is observed that in patients with higher BAFF levels in serum, the prognosis and survival are much worse [30].

Based on recent knowledge about genetic abnormalities in gastric MALT lymphoma, there is a model of multistep pathogenesis. On the background of chronic inflammation and antigenic stimulation occurs genetic instability. As a result, many possible translocation and unbalanced aberrations are observed. 

## 4. Symptoms and Diagnosis

The symptoms presented by patients with gastric MALT lymphoma are extremely unspecific. This causes difficulties with making final diagnosis and finding disease at an early stage. The signs of the disease are usually connected with involved location. Gastric MALT lymphoma can be long-time asymptomatic or associated with dyspepsia, abdominal pain, vomiting, diarrhea, obstruction, and nausea. Sometimes bleeding from gastrointestinal tract or even perforation may occur while extensive lesions are present. As a result, symptoms of anemia like paleness, weakness, or easy fatigue can be observed. B symptoms (weight loss, unexplained fever, and night sweats) in gastric MALT lymphoma are very rare, but the most common of the above is weight loss. A prompt diagnosis is crucial, but, unfortunately, it is usually made by incidence. Patients with early stage of disease have usually low tumor growth and minimal possibility to spread. The clinical course is indolent and there is a good response to the treatment. In contrast, patients with advanced stage of disease can undergo transformation to more aggressive lymphoma and may become resistant to treatment. 

Not only symptoms but also endoscopic picture can be inconclusive. Difficulties often arise to differentiate between chronic gastritis or ulcer from an early-stage lymphoma. In order to confirm the diagnosis, a histopathologic evaluation of the gastric biopsies is indispensable. Routine histology and immunohistochemistry are required to correctly distinguish the disease. There always must be made PCR or FISH analysis for t(11;18), which is important to separate groups that will not respond to standard treatment. Characteristic for gastric MALT lymphoma are lymphoepithelial lesions (LEL) with the presence of mainly two types of cells: neoplastic centrocyte-like or small lymphoid. Occasionally, there can be seen atypical plasmacytic tumor cells. There is no specific immunohistochemical profile typical for gastric MALT lymphoma diagnosis. In 50% of patients, there is coexpression of CD43/BCL2. Neoplastic cells are positive for CD-20 and negative for CD-10, CD-23, and cyclin D1.

Moreover, *Helicobacter pylori* infection must be investigated. If it is negative in histochemistry, rapid urea breath test or fecal antigen test have to be made. Another analysis to prove absence of *Helicobacter pylori* infection is serological test for CagA antibodies and *Helicobacter pylori*-IgG antibodies [31]. Sometimes, there is possibility to detect other *Helicobacter* species, for example, *heilmannii* or *felis* [32].

## 5. Staging and Risk Factors

Before taking any decision on how aggressive the treatment should be, it is extremely important to perform a complete staging of the disease. What is more important is that risk factors and individual parameters, which can affect later therapy, are crucial. Medical history must include information about the age, time of the first symptoms, the family history, and medical condition. The most important factor that we rely on during choosing method of treatment is clinical stage of the patient. During physical examination, it is important to remember about Waldeyer's ring, which is mandatory in every gastric lymphoma patient. Staging in gastric MALT lymphoma is similar to that in other types of lymphomas. According to recent European Society for Medical Oncology (ESMO) recommendations [33], it should include morphology with basic biochemical studies. If the blood cell count is lower, it can be caused by infiltration of bone marrow. Biochemical tests can detect liver or kidney problems, which can be important before the beginning of a chemotherapy. It can also detect mineral abnormalities which should be corrected before treatment. Lactate dehydrogenase (LDH) and *β*2-mikroglobulin are prognostic factors and will be abnormally high in patients with fast-growing tumor. Coagulogram is another important test which shows us if the blood is clotting properly. Every newly diagnosed patient should be examined in case of certain viral infections that can affect treatment, such as hepatitis B and C or human immunodeficiency virus (HIV). 

In every case, computed tomography (CT) scans of neck, chest, abdomen, and pelvis, which are crucial to evaluate enlarged lymph nodes, should be performed. Core needle biopsy of bone marrow is made to diagnose possible infiltration of neoplastic cells. It was confirmed that 15% of gastric MALT lymphoma patients have lymphoma cells in bone marrow. Positron emission tomography (PET) has still not confirmed clinical necessity, but it can be extremely helpful in controversial cases. Moreover, during staging procedures of gastric MALT lymphoma, gastroduodenal endoscopy must be made. Biopsies are taken from different sites of gastrointestinal tract (e.g., stomach, duodenum, and gastroesophageal junction) and every location that looks suspicious. 

There is no special staging scale for gastric MALT lymphomas. Most often, Ann Arbour staging is employed, which describe the extend of all types of non-Hodgkin lymphoma in adults. This classification was modified by Musshoff et al. [34]. Thus, staging of gastric lymphoma based upon the Ann Arbor system includes stage I E, which is disease limited to the stomach without nodal spread. Stage II E_1_ is tumor in the stomach with spread to adjacent contiguous lymph nodes. Stage II E_2_ is tumor in the stomach with spread to lymph nodes that are noncontiguous with the primary tumor. Moreover, if the spleen is affected, we add S. If the person has any of the B symptoms, we add letter B, and if is asymptomatic, we assign A (Table 1).

Prognostic factors in gastric MALT lymphoma are similar to the value for non-Hodgkin B-cells lymphoma. Factors that determine poor outcome are age, high level of LDH in serum, higher ECOG performance status, stages III and IV in Ann-Arbour scale, white blood count, and more than one extranodal site. It was observed that patients with nodal invasion has difficulty with complete remission after eradication treatment.

Some genetic abnormalities are thought to be bad prognostic factors. For instance patients, with t(11;18)(q21;q21) especially, are resistant to the first line therapy, and remission rate was lower than that in patients of *API2-MALT1 *negative (78% versus 22.2%; *P* = 0.0001) [35]. That is why followup is so important in this group. Only one study so far proved that the presence of t(3;14)(p14;q32) is connected with poor clinical outcomes of patients with gastric MALT lymphoma [25].

## 6. Treatment

While *Helicobacter pylori* plays a main role in the pathogenesis of MALT lymphoma, it is also crucial in approach to the treatment. According to current international guidelines, first line treatment for localized *Helicobacter pylori*-positive patients should be dual eradication therapy [36–38]. The treatment may be used with every highly effective antibiotics against *Helicobacter pylori*, taking into consideration the locally expected antibiotic resistance. If there is no response to the therapy above, second line triple or quadruple therapy is used. It was reported that after two lines of treatment, 99.8% of patients were cured from gastritis [39]. In a large study of 1408 patients, remission after eradication treatment in early stage was observed in 77.5%. 

Unfortunately, in 5%–10% of gastric MALT lymphoma patients, we cannot confirm *Helicobacter pylori* infection. Moreover, more than 30% patients are resistant to first line treatment, and 30% of them have t(11;18)(q21;q21). Treatment for this patients should be chosen individually depending on the clinical stage of disease. For those who have stable disease without any symptoms, the best approach is “watch and wait.” This approach will be valid for older patients with comorbidities. Potential risk factors like molecular markers should be taken into consideration as well. Aggressive therapy should be considered in symptomatic or progressive disease. Radiotherapy, chemotherapy, and/or surgery can be considered after unsuccessful eradication treatment. Further recommended therapy in this group has not been established so far. 

Surgery is considered to be a standard therapy in therapy of patients with gastric MALT lymphomas, but, recently, the value of this therapy has been not confirmed. Even if the lymphoma is localized at early stage, the gastrectomy should be rather extensive due to the nature of the disease. Sometimes further treatment is still required. Moreover, it is a major surgery and can be associated with serious complications and worsen a quality of life. German Multicenter Study Group (GMSG) presented no difference between survival in patients treated with gastrectomy compared to eradication (overall survival rate 82% to 84%) [40]. What is more important is that there were observed 50% long-term complications were observed after surgery [41].

In few studies, there was confirmed an excellent disease control by using radiotherapy. The use of a modest dose of involved fields was performed on resistant-to-eradication therapy patients with early-stage disease. The dose was 25–35 Gy to the stomach and perigastric nodes for the period of 4 weeks [42, 43]. Compared to surgery, no serious long-term complications and toxicity were observed. Only nausea and anorexia were present during the time of radiotherapy.

For a long time it was believed that gastric MALT lymphoma is just a localized disease and that surgery and radiotherapy are the best treatment strategy. Now, when it is well known that it is disseminated disorder chemotherapy, it became more important. Still, there are no standard recommendations for relapse or progressive patients after therapy and for those with late stage of the disease from the beginning. It was observed that chemotherapy alone is more effective than surgery apart from some cases with gastric obstruction [44]. Many chemotherapeutics are tested. The most commonly used are alkylating agents, nucleoside analogs in combination with corticosteroids. Complete remission (CR) after oral monochemotherapy with cyclophosphamide was 83% in a study by Nakamura and coworkers [45]. Unfortunately, patients with positive translocation t(11; 18) are resistant to second line therapy with oral monochemotherapy with alkylating agents. Nucleoside analogs are confirmed to be effective in treatment of different kinds of indolent lymphomas. A polychemotherapy with fludarabine and mitoxantrone (FM) has a very good effect on patients with gastric MALT lymphoma in both first and second line treatment. All groups that consisted of 20 people achieved complete remission [46]. Also the role of cladribine or 2-chlorodeoxyadenosine (2-CdA) was investigated. The complete remission after 4 cycles achieved 84% of investigated and all of them reacted to the treatment [47]. It is important that patients with translocation t(11;18) respond to therapy as well [48]. After 2-CdA, there were observed complications such as toxicities of 3 and 4 grade of WHO, mainly leukopenia, infections, and secondary neoplastic disease. There are highly effective drugs which should be individually considered in each patient.

Nowadays, immunotherapy became an extremely important part of treatment of non-Hodgkin lymphomas. The most commonly used is rituximab. It is a chimeric mouse/human monoclonal antibody specified to CD20 antigen expressed on the surface of B lymphocytes. Firstly, its effectiveness was shown in follicular lymphoma [56]. Now it is widely used alone or in combination with chemotherapeutic drugs in many types of B-cell non-Hodgkin lymphomas. Rituximab binds to CD20 antigen and activates the lysis of B cells by mediating cytotoxicity of complement dependent (CDC) and cell-mediated cytotoxicity antibody dependent (ADCC). It is also believed to induce cell death by apoptotic mechanism. The role of this drug is still not clear in gastric MALT lymphoma. In 2003, there was a first-phase study by Conconi et al. [57] with rituximab in monotherapy in patients at any stage. The CR was observed in 29% and overall response rate (ORR) was 64%. The toxicity of this treatment was moderate or even mild, but the relapse rate was 36%. An important fact is that patients with translocation t(11;18) are responsive to rituximab treatment [58, 59]. What is more important is that in a study by the International Extranodal Lymphoma Study Group (IELSG), it was confirmed that chlorambucil in combination with rituximab was more effective than chlorambucil alone [60]. Also in phase II clinical trial by Troch et al. CR by rituximab with cladribine was achieved by 58% of patients [54]. The conclusion is that rituximab may a benefit in individual patients, but for the majority it is not sufficient when used alone. It is more effective in combination with standard chemotherapeutics.

The efficacy of the combination of rituximab with chlorambucil was evaluated in a randomized study (comparator was chlorambucil alone) by the International Extranodal Lymphoma Study Group (IELSG) in gastric MALT lymphomas that had failed antibiotics and in nongastric MALT lymphomas. The preliminary report [40] showed that the 5-year event-free survival was significantly better for patients treated with chlorambucil plus rituximab. There were also studies by Raderer et al. with cycles generally used in more aggressive lymphomas. Twenty-six patients were administrated rituximab plus cyclophosphamide, doxorubicin or mitoxantrone, vincristine, and prednisone. Complete remission was observed in 77% and partial remission was achieved in 27% [53]. Lately, bortezomib, the first therapeutic proteasome inhibitor, was examined by Kiesewetter et al. in 2012 with CR in 33% and PR in 27.8% [55]. The results on phase II studies with chemotherapy and immunotherapy are shown in Table 2.

Still the place of autologous hematopoietic stem cell transplantation is unknown. So far, it is not a standard for treatment of indolent lymphomas. Outcomes in gastric MALT lymphoma patients with progressive, disseminated disease are very comparable with outcomes in follicular lymphoma.

## 7. Followup

Although gastric MALT lymphoma has a very favorable outcome, it is still important to have a proper followup. It is possible that the disease will return even after 5 years of complete remission. The relapse can be due to reinfection of *Helicobcater pylori*. In a study by Zullo et al., reinfection was observed in 2.7% [61]. The followup is obligatory in patients with gastric MALT lymphoma to identify early phase of the recurrence of the disease. To confirm a complete remission, there should be done both endoscopic and histological examination. Although, there are no specified recommendations for a followup, the biopsy of gastric sites should be made every 6 months in first two years, and later once a year for the next five years. Systemic followup consist of blood tests and minimal adequate radiological and ultrasound and should be made at least once a year in the first 5 years. The most common are chest X-ray and abdomen ultrasound. The transformation in more aggressive lymphoma is low at the level of 0.05% [35], but there is a higher risk of occurrence of secondary neoplasm [62] and gastric cancer [63]. These studies confirm that patients with gastric MALT lymphoma need a long-term followup not only to detect early recurrence but also to find secondary disease. 

## 8. Conclusions

Recently, enormous progress has been made in better understanding of pathogenesis of gastric MALT lymphoma. Many important chromosome aberrations, such as t(11;18), have been detected. It has a great influence on the development of new and more effective treatment strategy. There still remain cellular and molecular routes that need to be explored and clarified. Still, not enough clinical trials are performed due to rare expression and high effectiveness of first line treatment of gastric MALT lymphoma. What is more important is that early diagnosis of gastric MALT lymphoma is extremely important. While the symptoms are unspecific or not, always during the endoscopic exam the complete histological biopsies must be taken to make diagnosis correctly. The less advanced the stage of the disease, the bigger the chances to achieve complete remission.

## Figures and Tables

**Table 1:** Ann Arbour clinical staging for gastric lymphoma.

Stage	Localization
I E	Confined within the gastric wall
II E_1_	Involvement of stomach and contiguous lymph nodes
II E_2_	Involvement of stomach and noncontiguous subdiaphragmatic lymph nodes
III	Lymph nodes on both sides of the diaphragm
IV	Visceral metastasis or second extranodal site

Subscripts that can be added to staging:

E: extranodal, when lymphoid tissue outside lymph nodes is involved.

X: is added when the largest diameter is above 10 cm (called bulky disease).

S: is added when the spleen is involved.

A or B: B is added when one of B symptoms is present, and A is for asymptomatic patients.

**Table 2:** Chemo/immunotherapy lymphoma as a second line treatment in gastric MALT, phase II trials.

Authors	Treatment	*n*	CR	PR	SD
Nakamura et al. 2005, [45]	Cyclophosphamide	12	83%		17%
Raderer et al. 2005, [49]	Oxaliplatin	4	56%	38%	6%
Jäger et al. 2006, [50]	Cladribine	19	100%		
Martinelli et al. 2005, [51]	Rituximab	27	46%	31%	
Conconi et al. 2011, [52]	Bortezomib	13	46%	15%	31%
Zinzani et al. 2004, [46]	Fludarabine and mitoxantrone	20	100%		
Raderer et al. 2006, [53]	R-CHOP (15 patients) R-CNOP (11 patients)	26	77%	23%	
Troch et al. 2013, [54]	Rituximab and cladribine	40	58%	23%	13%
Kiesewetter et al. 2012, [55]	Lenalidomide	18	33%	27.8%	16.7%

*n*: number of patients, CR: complete response, PR: partial response, SD: stable disease.

R-CHOP: rituximab plus cyclophosphamide, doxorubicin, vincristine, and prednisone.

R-CNOP: rituximab plus cyclophosphamide, mitoxantrone, vincristine, and prednisone.
